# The Emerging Role for CTL Epitope Specificity in HIV Cure Efforts

**DOI:** 10.1093/infdis/jiaa333

**Published:** 2021-02-15

**Authors:** Clarety Kaseke, Rhoda Tano-Menka, Fernando Senjobe, Gaurav D Gaiha

**Affiliations:** 1 Ragon Institute of Massachusetts General Hospital, Massachusetts Institute of Technology, and Harvard, Cambridge, Massachusetts, USA; 2 Gastrointestinal Unit, Department of Medicine, Massachusetts General Hospital, Boston, Massachusetts, USA; 3 Virology Program, Harvard Medical School, Boston, Massachusetts, USA

**Keywords:** CTL, epitope, HIV, cure, vaccine, network

## Abstract

The development of an effective human immunodeficiency virus (HIV) cure is a critical global health priority. A major obstacle to this effort is the establishment of a latent reservoir of HIV infected cells, which necessitates lifelong therapy, causing both logistical and adherence burdens for infected individuals. However, in a subset of these individuals, cytotoxic T lymphocytes (CTLs) can durably suppress viral outgrowth in the absence of therapy, providing a path towards a viable HIV cure. In this review, we discuss the emerging role that CTLs have in HIV cure efforts, with particular emphasis on epitope specificity. Recent studies have demonstrated that successful in vivo containment of the virus is rooted in the specific targeting of fitness-constrained, mutation-resistant regions of the HIV proteome. We highlight these new insights, providing context with previous observations in HIV and other models of viral control, and delineate their translation into a therapeutic vaccine.

Despite over 3 decades of work, the human immunodeficiency virus (HIV)/AIDS epidemic continues to have enormous implications worldwide, with an estimated 37 million infected individuals [1]. While combination antiretroviral therapy (cART) has certainly curtailed the global burden of disease, the ability of the virus to establish a persistent latent reservoir deep within tissue sites requires that HIV-infected individuals remain on lifelong treatment [2]. Such therapy is a major logistical burden, particularly in low/middle income countries, and has been associated with substantial side effects, issues with drug adherence, and non-AIDS–related clinical morbidities [3]. Thus, there is a great need for the development of new modalities that can suppress the viral reservoir in the absence of cART and thereby obviate the need for lifelong therapy.

Among the various strategies under investigation are the induction of cytotoxic T lymphocytes (CTLs), which constitute a major component of the host immune response to HIV. The emergence of HIV-specific CTLs during acute infection coincides with a decline in plasma viremia [4, 5] and, more recently, CTLs were also shown to play a key role in suppressing viremia during treatment with cART [6]. However, perhaps more pertinent to HIV cure efforts, is the existence of a rare subset of individuals, known as HIV controllers, who have the capacity to naturally suppress the virus to undetectable levels in the absence of cART. Highly functional HIV-specific CTL responses play a key role in mediating the HIV controller phenotype [7–9], but the extent to which CTL epitope specificity influences viral control has only more recently come into view [10]. In this review, the increasing evidence in support of focusing CTL responses onto specific regions of the viral proteome for HIV cure efforts is discussed.

## HIV CONTROLLERS: A COMPELLING MODEL OF FUNCTIONAL HIV CURE

In the vast majority of individuals, untreated HIV infection leads to sustained viremia, CD4^+^ T-cell decline, and progression towards AIDS. However, for HIV controllers, the absence of therapy does not prevent these individuals from durably suppressing plasma viremia to below the transmission/progression threshold (2000 RNA copies/mL) or maintaining normal CD4^+^ T-cell counts, making them compelling natural examples of a functional drug-free HIV cure. While these individuals do have smaller viral reservoirs with lower sequence diversity [11, 12], studies have demonstrated that cells from HIV controllers are infectable and replication-competent virus is recoverable [13, 14], implicating a key role for the immune response in mediating viral suppression. Interestingly, there is a subset of HIV controllers, now termed “exceptional controllers,” who have such effective viral suppression that full-length replication-competent virus cannot be recovered, despite sampling of large numbers of peripheral CD4^+^ T cells and anatomically relevant tissues sites, such as the gastrointestinal tract [15]. These patients also demonstrate seroreversion, whereby anti-HIV antibody responses are no longer detected, a phenomenon typically reserved for individuals who have received early and prolonged cART [16]. Collectively, these cases demonstrate that durable, drug-free HIV remission can be achieved, further illustrating the need to define and translate the immunological underpinnings of HIV control to the broader HIV-infected population.

## THE GENETIC BASIS OF HIV CONTROL: HLA/MHC-BASED ASSOCIATIONS

Initial cohort studies of HIV-positive individuals first revealed the impact of human leukocyte antigen (HLA) class I molecules on time to AIDS progression [17], and subsequent studies revealed a striking enrichment of the specific allele HLA-B*57 in HIV long-term nonprogressors [18]. Both of these studies clearly implicated a key role for HIV-specific CTLs in viral control, given the established function of major histocompatibility complex (MHC)/HLA class I molecules to present virally derived peptides on the surface of infected cells for CTL recognition and killing. Genome-wide association studies (GWAS) confirmed that the strongest determinants of viral load were the HLA-B*5701 allele in individuals of European ancestry [19], and the closely related HLA-B*5703 allele in African-Americans [20], although it is important to note that these specific HLA alleles were neither necessary nor sufficient for viral control. A separate GWAS study comparing over 974 HIV controllers and 2648 progressors further delineated protective (B*5701, B*5201, B*2705, B*1402) and risk (B*07, B*08, B*35) alleles but, more importantly, offered compelling new insight by revealing that specific residues within the HLA molecule that line the peptide binding groove (residues 67, 70, and 97) were even more strongly associated with viral control than any individual HLA allele [21]. This suggested that the link between HLA and HIV control could potentially be attributed to the specific epitopes presented by these alleles for recognition by CTLs.

## ELITE CONTROL OF MURINE PICORNAVIRUS: A MODEL OF MHC ASSOCIATION AND CTL EPITOPE SPECIFICITY

To further investigate the underlying immunologic mechanisms that govern control and persistence of chronic viral infections, mouse models have been frequently employed given the inherent constraints of studying human subjects. Among the most commonly used is the lymphocytic choriomeningitis virus (LCMV), in which infection with 2 viral clones, LCMV Armstrong or LCMV Clone 13, leads to either acute viral clearance by CTLs or a persistent viral infection, respectively [22]. Numerous studies have leveraged this marked difference in disease outcome to examine the unique cellular pathways and transcriptional networks that emerge within persistent antigen-exposed CTLs from Clone 13 infected animals [23–25], which have subsequently been validated in samples from chronically HIV-infected individuals [26, 27].

While these studies have certainly revealed key insights into the CTL dysfunction that can develop during chronic viral infection, they have been less useful in determining possible causes of viral control. This is in part due to the fact that LCMV Clone 13 uses a distinct mode of viral persistence whereby its high-affinity surface glycoprotein GP1 leads to preferential infection and dysfunction of dendritic cells, and consequently weak CTL induction [28, 29], which is not observed in early and acute HIV infection [5]. In contrast, the relatively understudied Theiler’s murine encephalomyelitis virus (TMEV) offers a far better murine model for HIV control, given the marked differences in virus-induced pathologies between mice with distinct MHC class I haplotypes [30]. Similar to findings of HIV controller and progressor GWAS studies, differences in susceptibility to TMEV-induced demyelination and inflammation were also mapped to the MHC locus, and linked to the expression of specific murine *H-2D* class I alleles that conferred either resistance or susceptibility [31]. Further studies demonstrated that mice susceptible to TMEV infection could become resistant upon transgenic introduction of the protective *H-2D*^*b*^ gene [32], followed by the development of an early and abundant TMEV-specific CTL response [33]. This *H-2D*^*b*^-mediated CTL response was ultimately revealed to be primarily directed against a single immunodominant epitope derived from the TMEV viral protein 2 [34]. This indicates that successful viral control of a murine picornavirus, which has a clear MHC association in close similarity to HIV control, was due to the presentation and targeting of a specific viral epitope by CTLs. Further studies indicated that this particular epitope was highly resistant to mutation given its role mediating key interface interactions in the TMEV picornavirus coat [35], indicating a potentially broad feature of CTL epitopes that mediate viral control.

## VIRAL SEQUENCE ANALYSIS: FURTHER IMPLICATING CTL EPITOPE SPECIFICITY IN HLA-ASSOCIATED HIV CONTROL

Initial steps to evaluate the role of CTL epitope specificity in HIV control examined whether the targeting of certain viral proteins by CTLs had a more beneficial effect on viral suppression than others. A large study of chronically infected individuals found that broad, interferon-γ ^+^ CTL responses specific for Gag were associated with lower HIV viral loads, while similar responses specific for the viral envelope proteins were found in individuals with higher viral loads [36]. Given the relative sequence conservation of Gag in comparison to envelope, it was presumed that sequence conservation would be a defining feature of protective CTL epitopes. However, a study evaluating the targeted CTL epitopes of controllers and progressors across multiple proteins in HIV found no significant difference in viral sequence conservation [37].

While this finding was somewhat unexpected, it is important to note that sequence conservation is an imperfect measure of mutational resistance. Analysis of conserved element vaccines revealed that only a subset of conserved residues exacted a high fitness cost upon mutation [38, 39], while comprehensive mutation of a conserved HLA-A2–restricted HIV epitope revealed that the majority of variants have only modest effects on viral replication [40]. This revealed that viral sequence conservation analysis was not an adequate proxy for mutational constraint, in part due to its inability to capture the interdependencies between amino acid residues. Thus, higher order sequence analyses of couplings between viral residues were employed for Gag using random matrix theory [41] or quantitative fitness landscapes [42]. This demonstrated that constraints on viral sequence evolution were multidimensional in nature, and likely rooted in the interaction of amino acids within the native 3-dimensional structure of viral proteins and protein assemblies. Moreover, multidimensional sequence-constrained Gag residues were preferentially presented by protective HLA alleles, and had costly effects on viral replication when mutated.

## STRUCTURE-BASED NETWORK ANALYSIS: A NEW METHOD TO EVALUATE THE ROLE OF CTL EPITOPE SPECIFICITY IN HIV CONTROL

These insights from the elite control of TMEV and multidimensional sequence analysis of HIV suggested that further evaluation of HIV protein structure could ultimately distinguish protective from nonprotective CTL epitopes. However, a major limitation was the absence of available tools to quantitatively evaluate structural constraints. Traditional structural analyses provided insight into the fine detail of local amino acid residue interactions, but were unable to reveal the importance of individual residues to the global architecture of the protein—a key feature of evolutionary constraint [43].

**Figure 1.**
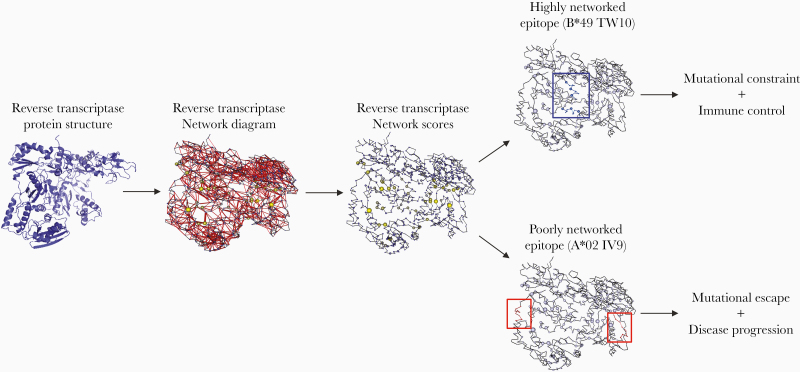
Application of structure-based network analysis to HIV protein structures to delineate mutationally constrained CD8^+^ T-cell epitopes. Structure-based network analysis utilizes atomic-level protein crystal structure data, in this case for HIV reverse transcriptase (PDB: 3KJV), to construct a protein network by calculating and summing all noncovalent interactions between residue side chains. The width of the red edges indicates interaction strength. The protein network is then used to calculate network scores for each amino acid residue, indicating its topological importance to the overall structure of the protein (node size indicates relative network score). Using these scores, linear CD8^+^ T-cell epitopes can then be quantified on the basis of their topological importance as either highly networked or poorly networked. Recent work has demonstrated that CTL targeting of highly networked epitopes is associated with immune control, while targeting of poorly networked epitopes is associated with disease progression [44].

In order to address this shortcoming, a recent study applied network analysis metrics to protein structure data to define the global importance of individual amino acid residues and CTL epitopes in the HIV proteome (Figure 1) [44]. This was accomplished by using atomic-level coordinate data from the Protein Data Bank to build networks of amino acid residues (nodes) and noncovalent interactions (edges), which included van der Waals interactions, hydrogen bonds, salt bridges, disulfide bonds, pi-pi interactions, pi-cation interactions, metal coordinated bonds, and local hydrophobic packing. These protein network-based representations were then analyzed by an array of network centrality metrics in order to compute a quantitative network score that captured the relative contribution of each amino acid residue to the protein’s topological structure.

The approach was validated on non-HIV proteins of known structure and function, which demonstrated highly significant inverse correlations between amino acid network score and experimentally determined mutational tolerance. This confirmed the value of the network score as a proxy for mutational resistance. Moreover, network score outperformed sequence conservation and relative solvent accessibility in its capacity to define mutation-resistant residues. Thus, the approach was applied to the HIV proteome and revealed that network score could distinguish residues with similar sequence conservation, but with disparate effects on viral fitness when mutated. Additionally, it demonstrated that the interconnectedness of residues in Gag p24 was significantly higher than other proteins, such as envelope, potentially providing a biophysical explanation as to why increased CTL breadth to Gag is associated with better control of viral replication.

In the context of HLA associations with HIV control, the network approach revealed that protective HLA alleles preferentially presented epitopes comprised of residues with high network scores (highly networked epitopes), while risk HLA alleles rarely, if ever, presented such epitopes. These observations indicated that differences in protective/risk HLA allele odds ratios could potentially be explained by the stochastic probability of targeting a highly networked epitope [21]. Furthermore, HLA-B*57 was found to present an inordinately high number of highly networked epitopes, which includes the commonly targeted KF11 epitope (Gag p24 30–40) that has limited pathways of CTL escape [45], possibly explaining why this HLA allele is consistently enriched among HIV controllers. Moreover, when the 20 distinct amino acids were ranked by network score, tryptophan emerged with the highest mean score due to its frequent presence within the hydrophobic cores and oligomeric interface regions of the HIV proteome. This is pertinent as tryptophan is also a common carboxy-terminal HLA anchor residue for B*57-restricted epitopes but is only rarely utilized by other HLA alleles.

Interestingly, while median network score of presented epitopes differentiated protective and risk HLA alleles, several epitopes restricted by neutral HLA alleles were also found to be highly networked. This finding provided the opportunity to investigate whether HIV control could be decoupled from its association with HLA alleles, and instead linked to the targeting of highly networked epitopes by functional CTL responses. Indeed, evaluation of proliferative HIV-specific CTLs in a cohort of HIV-positive individuals with diverse clinical phenotypes revealed preferential targeting of highly networked epitopes by HIV controllers, but only rare and weak targeting of these epitopes by progressors, irrespective of HLA allele [44]. In addition, plasma virus sequencing demonstrated that robust targeting of highly networked epitopes by CTLs resulted in nearly negligible sequence variation, particularly at HLA anchor and T-cell receptor contact sites [44]. Thus, the unique perspective provided by structure-based network analysis identifies specific and functional CTL targeting of highly networked epitopes, which exhibit mutational resistance in the presence of strong CTL pressure, as a broad mechanism of HIV control, and thereby provides a path towards an HIV cure.

## TRANSLATION OF HIGHLY NETWORKED EPITOPES TOWARDS A DELIVERABLE ENTITY FOR PATIENTS

The identification of highly networked epitopes that are presented by nonprotective HLA alleles, but which constitute major HLA supertypes (eg, HLA-A*02, A*03, B*07) [46], provides the putative basis for the rational design of a broadly effective, therapeutic CTL-based HIV vaccine. Notably, a number of highly networked epitopes from Gag p24 (TL9, KF11, QW9) were found to be “unmutated” in the latent reservoir of chronic ART-treated individuals, and facilitated suppression of viral outgrowth when targeted by CTLs [47]. However, important next steps include the design of an immunogen that incorporates a sufficient number of highly networked epitopes for broad coverage of the population, while limiting the inclusion of immunodominant, nonprotective epitopes, which can diminish the induction of protective responses [48]. In addition, the selection of the appropriate delivery modality is critically important, given the need to shift CTL immunodominance hierarchies towards subdominant highly networked epitopes. Studies evaluating a chimpanzee adenovirus vector prime and a modified vaccinia Ankara boost of a conserved element vaccine (tHIVConsv) led to a clear shift in CTL specificity towards conserved regions [49, 50], although this immunogen is comprised of a combination of highly networked and poorly networked epitopes. Nonetheless, these data provide evidence that redirection of CTL responses towards focused regions of the HIV proteome is possible by therapeutic vaccination, and could therefore be reasonably utilized to exclusively direct CTLs to highly networked epitopes.

## CONCLUDING REMARKS

With renewed interest in T-cell–based cure strategies for HIV, it is vitally important that careful consideration is given to the specificity of CTL responses. Systematic study of HIV controllers has revealed that enrichment of specific HLA class I alleles within these individuals is likely the result of preferential presentation and functional CTL targeting of epitopes with higher-order sequence constraints, due to their presence at topologically important, mutation-resistant regions of the viral proteome. Moreover, the ability now to decouple HIV control from these specific HLA class I alleles and link to specific CTL epitopes provides a key step towards clinical translation. Developing a suitable therapeutic vaccine candidate that leverages these findings towards a functional HIV cure is an important next step.

## Notes

***Financial support.*** This work was supported by the National Institutes of Health, National Institute of Allergy and Infectious Diseases (grant number K08 AI140960 to G. D. G.); Burroughs Wellcome Career Award for Medical Scientists (to G. D. G.); and the Bill and Melinda Gates Foundation (to G. D. G.).

***Potential Conflicts of Interest.*** G. D. G. has a patent Multi-Networked Epitope Vaccine pending. All other authors report no potential conflicts. All authors have submitted the ICMJE Form for Disclosure of Potential Conflicts of Interest. Conflicts that the editors consider relevant to the content of the manuscript have been disclosed.

Presented in part: Harvard University Center for AIDS Research Meeting Promising Approaches to HIV Remission and Cure, Cambridge, MA, 23 October 2019.
